# Elective Hand Surgery in Patients With History of Axillary Node Dissection: Risks and Patient Education

**DOI:** 10.7759/cureus.27461

**Published:** 2022-07-29

**Authors:** Muhammad Usman Awan, Gary Schwartz, Anastassia Shifchik, Skylar Harmon, Tatevik Malisetyan

**Affiliations:** 1 Allopathic Medicine, Nova Southeastern University Dr. Kiran C. Patel College of Allopathic Medicine, Fort Lauderdale, USA; 2 Anatomy, Nova Southeastern University Dr. Kiran C. Patel College of Allopathic Medicine, Fort Lauderdale, USA

**Keywords:** lymphedema, post operative complication, patient counseling, breast cancer, axillary lymph node dissection (alnd)

## Abstract

Objective: To determine if patients with a prior history of axillary lymph node dissection (ALND) secondary to breast cancer surgery and other procedures are at an increased risk of postoperative complications including lymphedema and infection following elective upper extremity surgery. Furthermore, the study aimed to evaluate the extent of patient education regarding lymphedema as a possible complication of upper extremity surgery.

Methods: A review of 312 patients presenting to the clinic with upper extremity pathologies was performed of which 15 patients had a history of surgeries secondary to breast cancer and 297 had no prior history of breast cancer. Nine out of 15 patients with prior breast procedures and 66 out of 297 patients with no such history underwent elective hand surgeries, with 22 out of the 75 patients having a history of ALND. Incidences of postoperative complications including lymphedema and infection were recorded. Afterward, a survey inquiring about patient education was conducted to assess whether the patients were educated regarding lymphedema and if so, when and from whom they received the counseling.

Results: No patients with a prior history of ALND secondary to breast cancer or other surgeries developed a postoperative infection or onset of lymphedema, and no patients with preoperative lymphedema had any worsening of lymphedema or infection postoperatively. The survey conducted afterward revealed that 61% of the patients with a prior history of breast cancer-related procedures including lymph node dissection were never counseled regarding lymphedema as a possible complication of hand surgery. Furthermore, 75% of the survey participants wished they were given more information about possible causes and complications of, and ways to prevent or minimize the possibility of lymphedema developing postoperatively.

Conclusion: Prior history of ALND did not make patients more susceptible to postoperative complications, thus a history of isolated ALND or breast cancer surgery including ALND should not preclude elective hand surgical procedures from being performed ipsilaterally. Additionally, improvements in the degree of patient counseling regarding postoperative complications following hand surgery are needed as increased patient education is shown to be associated with a lower rate of complications and faster recovery times.

## Introduction

Lymphedema is the accumulation of fluid in tissues due to a failure of the lymphatic system drainage [1]. Patients who have undergone surgical procedures related to the breast are generally advised against elective hand surgery in the ipsilateral upper extremity due to the development or exacerbation of lymphedema. Additional elective surgeries can present an increased risk of an inflammatory response to an already compromised lymphatic system [2-3]. Guidelines advising against elective hand and upper extremity surgery due to lymphedema complications have since been revisited following their establishment in the 1970s [4-7]. Previous precautions have come into question with the development of surgical procedures and techniques. Less invasive surgical procedures have demonstrated lower risk compared to invasive surgical approaches [8-10]. Modern-day breast cancer treatments have demonstrated a lower risk of lymphedema with the introduction of breast-conserving surgery and less invasive lymph node dissection and irradiation.

A review of the literature provides very little data to support the theory of morbidity in the axillary dissected arm after surgical or medical procedures [7]. In patients without a history of ipsilateral lymphedema, complications following elective surgery were similar in those with a history of breast cancer and those without. However, there was evidence of a transient worsening lymphedema risk in patients with a history of lymphedema, though inconclusive due to the low sample size [11]. Lymphedema exacerbation and complications were not associated with tourniquet use. 

One of the most common causes of lymphedema within the United States is breast cancer surgery [12]. As of 2007, McLaughlin et al. established rates of lymphedema in 16% of patients following sentinel lymph node biopsy with axillary lymph node dissection (ALND) versus lymphedema in 5% of patients following biopsy alone [13]. A survey completed among health care professionals in 2010 asked if ipsilateral hand surgery following ALND was contraindicated. Of the respondents, 41% of hand surgeons stated a contraindication with the most commonly cited reason being an increased risk of lymphedema following surgery. Comparatively, 58% of hand surgeons reported that they would still operate on breast cancer patients post-ALND [14]. There continues to be debate over elective hand surgery in women who have had ALND despite the high incidence of breast cancer within the United States. 

Patient counseling and their level of perceived knowledge have shown to be associated with reduced incidence of postoperative complications [15]. Sufficient patient counseling regarding post-operative care including exercises and wound care techniques is especially important as a substantial number of postoperative complications occur at home [16]. Thus, counseling regarding lymphedema in post-ALND patients by a trained professional can serve an important role in reducing postoperative morbidity, helping reduce the recovery times. Patient counseling for the prevention of lymphedema can include but is not limited to at-home techniques including manual lymphatic drainage (MLD), compression therapy, and certain exercises [17].

MLD is used to increase lymphatic drainage by increasing rhythmic contractions of the lymphatics and is done through stationary circular strokes, scoop technique, pump technique, and rotary technique. Compression therapy, which involves wrapping the extremity with tight bandages or cloth sleeves, increases interstitial pressure in subcutaneous tissue thus preventing fluid outflow from capillaries and helping increase venous return [18]. Home-based rehabilitative exercise plans for the upper extremity can also help significantly reduce postoperative lymphedema as lymph fluid is propelled and drained through muscle contractions [19].

The purpose of this study was to determine if the risk of postoperative complications following elective hand surgery for conditions producing a significant functional loss in patients with a history of axillary node dissection due to breast cancer-related surgeries or other procedures is any higher or lower than those who have not undergone lymph node dissection. The study also aimed to assess the prevalence of patient counseling regarding lymphedema as a postoperative complication.

## Materials and methods

A retrospective chart review of 312 patients was performed to assess lymphedema complications and patient knowledge of complications and post-surgical care instructions. The review selected patients based on a history of breast cancer and prior ALND surgical history, including individuals with subsequent hand surgeries. Inclusion criteria for the study consisted of patients who opted for elective hand surgery and the exclusion criteria consisted of patients with prior-ALND that received elective hand surgery on the contralateral arm. Patients who underwent non-elective hand surgeries were also excluded.

The patients originally presented to the attending physician for upper extremity pathologies between April 2001 and September 2002. Of the 312 selected patients, 297 had no history of breast cancer and 15 had a history of breast cancer. 60% (N=9) of the breast cancer patients had surgery while 22.5% (N=66) of the non-breast cancer patients had surgery. There were two known cases of preoperative lymphedema. Standard operating procedures were noted on all patient surgeries including the use of intravenous (IV) regional anesthesia with an average tourniquet time of 26 minutes and tourniquet pressure of 250 mmHg. Only patients with a history of mitral valve prolapse or other medical conditions warranted preoperative antibiotics. 

All patients with a prior surgical history have had at least an ALND. The majority of the patients had ALND in conjunction with breast cancer surgeries including mastectomy (N=9), lumpectomy (N=3), and bilateral mastectomy (N=2) while N=8 had isolated ALND with other surgical procedures. Hand surgeries performed on patients included carpal tunnel release (40%, N=30), trigger finger release (53.3%, N=40), and DeQuervain’s (tenosynovectomy of the first dorsal compartment of the wrist) (6.67%, N=5).

A survey was conducted including all the aforementioned patients which evaluated the patient’s knowledge of lymphedema as a complication of hand surgery. Patients were asked to complete a survey following their treatment that assessed their knowledge of surgical complications. Their source of counseling regarding lymphedema, exercising, information about complications, and timing of post-surgical complications was evaluated in these surveys. Of the involved patients, 34% had a previous history of lumpectomy with lymph node dissections, 45% had a mastectomy with lymph node dissections, and 21% had other surgeries with lymph node dissections. Surveys were subsequently compiled and analyzed to assess the extent of patient counseling regarding lymphedema and the resources used for patient education.

## Results

**Figure 1:**
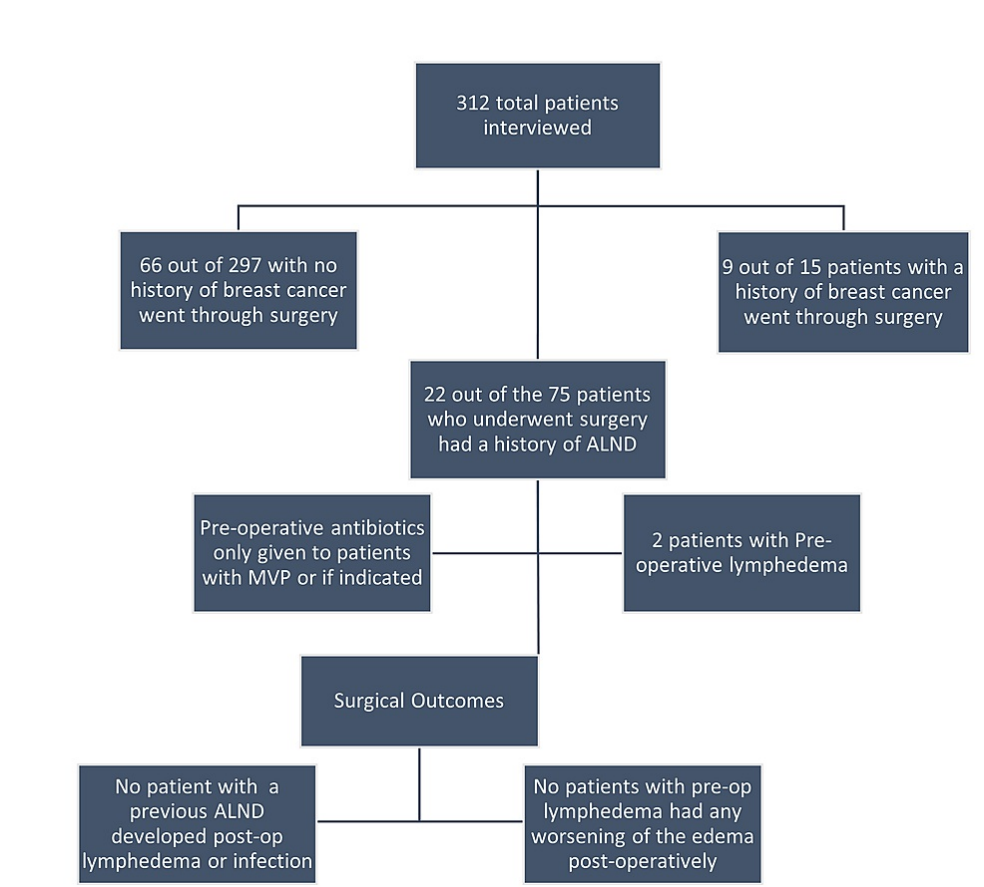
Breakdown of the patient population selected for the study ALND: Axillary Lymph node dissection; MVP: Mitral Valve Prolapse

A review of 312 patients' charts was completed out of which 75 patients opted for elective hand surgery. Twenty-two out of the 75 patients undergoing surgery had a prior history of ALND and two patients had pre-operative lymphedema. Surgical outcomes were recorded and indicated that no patient with pre-operative lymphedema had any worsening of the lymphedema post-operatively and no patient with a history of ALND developed lymphedema. There were no incidences of post-operative infection as well (Figure 1). 

**Figure 2:**
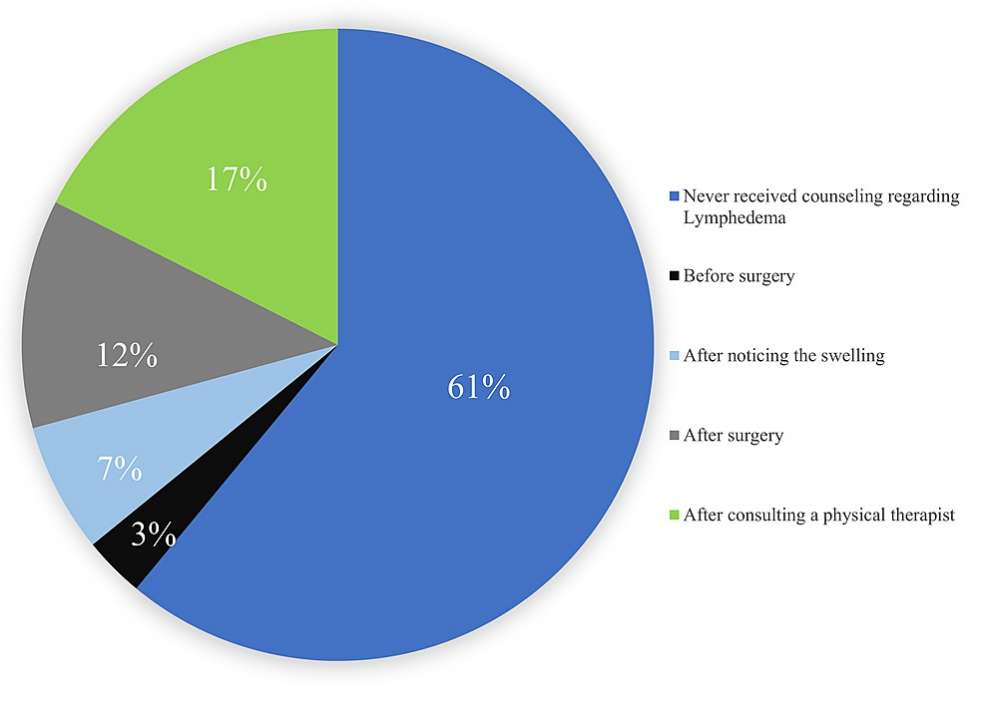
Breakdown of patient counseling regarding lymphedema

A survey conducted to assess the extent of patient education regarding lymphedema as a possible complication of hand surgery in post-ALND patients depicted that 61% (N=46) of the respondents were never counseled about lymphedema as a possible complication. Of those counseled, most reported counseling following the physical therapist consultation (17%, N=13). Of the rest, 12% (N=9) reported counseling after the surgery, 7% (N=6) after noticing the initial swelling, and 3% (N=2)reported being informed about it before surgery (Figure 2). The method of receiving information regarding lymphedema was also investigated with 40% (N=30) reporting their source being brochures, 22% (N=17) reported during physician follow-up appointments, 34% (N=25) in session with a physical therapist, 22% (N=17) in a seminar/lecture before surgery, and 15% (N=11) in a seminar/lecture after surgery.

**Figure 3:**
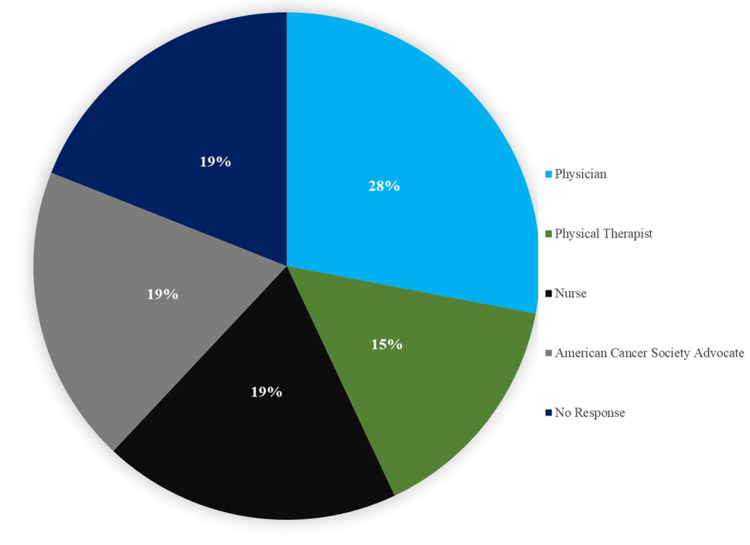
Source of patient counseling regarding post-op care

Additionally, 72% (N=54) of the total survey respondents stated that their respective surgical procedure had been adequately explained to them and 57% (43) reported that they were counseled on the necessary post-operative care and precautions. In regards to timing, 8% (N=6) of the respondents reported receiving the aforementioned counseling before the procedure and 50% (N=38) reported receiving counseling after the surgery, 42% (N=31) did not respond. The source of the post-operative care and precaution counseling was investigated and 28% (N=21) reported being counseled by their physician, 15% (N=12) by their physical therapist, 19% (N=14) by a nurse, 19% (N=14) by an American Cancer Society advocate, and 19% (N=14) did not respond (Figure 3). Inquiry regarding when patients would have liked to receive this information, 60% (N=45) reported before surgery, 23% (N=17) after surgery and 17% (N=13) did not answer the question. Lastly, 75% ( N=56) wished they were given more information about possible causes, complications, and ways to minimize the possibility of lymphedema developing post-operatively.

## Discussion

The history of ALND has been shown to increase the risk ratio of arm lymphedema when compared with individuals who do not undergo ALND [20]. Thus, avoiding elective hand surgery has become common practice in post-ALND patients due to a fear of worsening lymphedema and other potential complications, including delayed wound healing and infection. Patient concerns in regard to this matter are heightened when they are advised in this regard by their physicians [21].

Despite this, a review of the literature provides very little data to support the theory of morbidity in the axillary dissected arm after surgical or medical procedures. In this study, we demonstrate that elective hand surgery, when performed with strict sterile technique and surgical precautions, does not worsen edema postoperatively or lead to the development of infections. 

Of the 312 patients who completed the study, none with a previous ALND developed a postoperative infection. Patients with pre-operative edema did not have any worsening of the edema following surgery (Figure 1). The infection rate for the entire patient population was 0%. 

Similar results were seen in further studies. A 2015 study by Lee et al. completed a review of patients presenting to their hand clinic between 1998-2011 with previous breast cancer or melanoma with subsequent axillary node dissection [22]. Of the fifteen patients who were surveyed post-operatively (range 1 to 11.9 years following surgery), no new cases of lymphedema were found with no disease exacerbation in three patients with pre-operative lymphedema [22].

Another prospective study conducted by Gaston et al. (2016) depicted an analogous correlation between postoperative lymphedema in patients who underwent elective hand surgery and had a history of ipsilateral lymph node dissection secondary to breast cancer. Of the 44 patients who completed the follow-up appointments for the study, only one was found to have lymphedema at the two-week mark and there were no cases of lymphedema at the three months and six-month mark [23]. Thus, data from this study and previous studies suggest that the risk of lymphedema or infection should not preclude patients with a history of ALND from undergoing elective hand surgery for conditions that have significant symptoms including pain and functional loss.

Due to the high incidence of postoperative complications occurring at home [16], increased patient education is shown to have an influence on reducing postoperative morbidity [15]. Thus, it is important to properly educate patients regarding postoperative complications and the proper care techniques to deal with them. Additionally, Bastable reported higher patient education to be associated with increased patient empowerment and better adherence to the plan of care which leads to better long-term outcomes [24]. Given the importance of patients’ involvement in their care, the patient’s knowledge of lymphedema as a complication of hand surgery was evaluated.

Following surgery, a survey was conducted that evaluated the patient’s knowledge of lymphedema as a complication of hand surgery. Of these patients 34% had a lumpectomy with lymph node dissection, 45% had a mastectomy with lymph node dissection, and 21% had other surgeries with lymph node dissection previously.

In regard to lymphedema specifically, 61% of patients were never informed about it as a possible complication (Figure 2). The results could potentially be explained due to changing perspectives regarding lymphedema being considered a major postoperative complication. However, there is no clear pattern for when patients are likely to receive this information if they do. 

Following surgery, patients were given the opportunity to report when they would have liked to receive information regarding lymphedema risk with 60% reporting they would have preferred pre-operative education and 23% post-operative education. Of the respondents, 75% reported they would have appreciated more information about possible causes, complications, and ways to minimize the risk of lymphedema developing post-operatively. 

Given that only 8% of patients received information about lymphedema pre-operatively compared to the 60% that reported they would have liked to, this denotes a need for deeper patient involvement in care and thus further information on surgical complications prior to surgery. This is especially important as the initiation of patient education pre-operatively has been shown to reduce patient anxiety and the rate of peri and post-operative complications [25]. Moving forward, it is important to integrate patient education regarding possible complications and care procedures in the pre-operative workup.

Included in follow-up care instructions were post-operative care recommendations. This is especially important when it comes to lymphedema as there are multiple techniques including manual lymphatic drainage (MLD), compression therapy, and rehabilitation exercise programs that can be performed at home and have been shown to significantly reduce post-operative lymphedema [17]. Proper performance of the aforementioned techniques requires counseling from a trained professional yet our data demonstrated a wide source distribution of post-operative care information (Figure 3).

Patient education regarding post-operative care techniques was given pre-operatively to 9% of patients and post-operatively to 50% of patients. Post-operative care recommendations were only recognized by half of the patient population and the sources of the information varied widely with physical therapists and physicians making up only 45% of the cohort. This indicated an area of oversight that can have detrimental impacts on patient healing and recovery times as adherence to a proper at-home exercise plan has shown to significantly reduce lymphedema [19].

One of the main limitations of this study is the collection point of the data as it may demonstrate outdated practices when it comes to patient education from physicians regarding postoperative complications and care instructions. Thus, another study regarding patient counseling now can be done to assess any shift in clinical practices. Additional limitations of the study include the wide distribution that can be seen in many survey question responses which can be due to the format of the survey questions not being specific enough which can lead to respondents interpreting the survey questions differently. This prevents the establishment of strong correlations based on the collected survey data. Lastly, the smaller sample size of the study also poses a limitation as it can lead to less statistically significant results.

## Conclusions

Based on patient outcomes, the type of breast surgery or extent of axillary dissection did not make the patient more susceptible to complications. If strict sterile techniques, anesthetic, and surgical precautions are observed, a history of axillary dissection should not preclude elective hand surgical procedures from being performed. However, in terms of patient education, there was a wide range of education distribution. There was a discrepancy in what and when information was received by patients compared to when patients would have preferred to be informed. It is, however, unknown as to the impact of this information on the surgical healing of this cohort. As such, further research would need to be performed to follow patients through the effect of preoperative and postoperative patient education. Also, given the collection point of this data, an updated comparison of current patient education processes could be conducted. This could demonstrate an improvement in the steps undertaken to inform patients of upper extremity surgery complications.
